# BMP and Notch Signaling Pathways differentially regulate Cardiomyocyte Proliferation during Ventricle Regeneration

**DOI:** 10.7150/ijbs.59648

**Published:** 2021-05-27

**Authors:** Wenyuan Wang, Ye-Fan Hu, Meijun Pang, Nannan Chang, Chunxiao Yu, Qi Li, Jing-Wei Xiong, Yuanyuan Peng, Ruilin Zhang

**Affiliations:** 1 School of Life Sciences, Fudan University, Shanghai, China.; 2 Department of Medicine, Li Ka Shing Faculty of Medicine, The University of Hong Kong, Pokfulam, Hong Kong, China.; 3 School of Biomedical Sciences, Li Ka Shing Faculty of Medicine, The University of Hong Kong, Pokfulam, Hong Kong, China.; 4 Institute of Molecular Medicine, Beijing Key Laboratory of Cardiometabolic Molecular Medicine, Peking University, Beijing, China.; 5 School of Basic Medical Sciences, Wuhan University, Wuhan, China.

**Keywords:** heart regeneration, BMP signaling, Notch signaling, cardiomyocyte proliferation, cell-cycle arrest

## Abstract

Adult mammalian hearts show limited capacity to proliferate after injury, while zebrafish are capable to completely regenerate injured hearts through the proliferation of spared cardiomyocytes. BMP and Notch signaling pathways have been implicated in cardiomyocyte proliferation during zebrafish heart regeneration. However, the molecular mechanism underneath this process as well as the interaction between these two pathways remains to be further explored. In this study we showed BMP signaling was activated after ventricle ablation and acted epistatic downstream of Notch signaling. Inhibition of both signaling pathways differentially influenced ventricle regeneration and cardiomyocyte proliferation, as revealed by time-lapse analysis using a cardiomyocyte-specific FUCCI (fluorescent ubiquitylation-based cell cycle indicator) system. Further experiments revealed that inhibition of BMP and Notch signaling led to cell-cycle arrest at different phases. Overall, our results shed light on the interaction between BMP and Notch signaling pathways and their functions in cardiomyocyte proliferation during cardiac regeneration.

## Introduction

Myocardial infarction (MI) is a leading cause of death worldwide 1, 2. In adult mammals, the heart, as one of the least regenerative organs, replaces the infarcted myocardium with noncontractile scar tissue 3. On the contrast, zebrafish are capable to completely regenerate the injured hearts 4-6. New cardiomyocytes have been shown to arise from proliferation of pre-existing cardiomyocytes through genetic fate-mapping experiments 7, 8. Though natural cardiomyocyte turnover rate is low in adult humans 9, 10, accelerating such process after acute insult is therapeutically valuable. Thus, utilizing cellular and genetic factors that stimulate cardiomyocyte proliferation may provide viable solutions for promoting human cardiac regeneration.

Zebrafish cardiac regeneration involves multiple signaling pathways 11-13. BMP signaling is vital for vertebrate cardiovascular development 14, 15, and its function in cardiac injury and repair has become more appreciated. Since inhibitory effects of BMP2 on fibroblast function have been reported 16, exogenous BMP2 reduced the size of infarcted tissue by diminishing apoptotic cardiomyocytes in the border zone in a mouse permanent coronary occlusion model 17. While BMP4 enhances apoptosis and hypertrophy of cultured mammalian cardiomyocytes 18, BMP7 signaling attenuates myocardial fibrosis by inhibiting TGF-β responses in a rat MI model 19. BMP10 has been demonstrated to enhance inflammation activity after MI and exogenous BMP10 results in cardiomyocyte cell-cycle re-entry 20. Thus, BMP signaling has been suggested as a good candidate pathway for modulating cardiac regeneration 21, but its role in cardiomyocyte proliferation requires further investigation.

Notch signaling pathway also plays important roles in cardiac regeneration 22, 23. Notch signaling is activated in the endocardium of injured zebrafish hearts and its inhibition impedes cardiomyocyte proliferation and heart regeneration 6, 24-26. Endocardial Notch signaling has been shown to promote myocardial proliferative events through inducing BMP10 expression in adjacent cardiomyocytes 27-29. However, the interaction between these two pathways and their differential regulation of cardiomyocyte proliferation remains elusive. In this study we utilized the zebrafish larval ventricle ablation model and a cardiomyocyte-specific FUCCI (fluorescent ubiquitylation-based cell cycle indicator) system to tackle these questions. We demonstrated the epistatic relationship between BMP and Notch signaling pathways during ventricle regeneration and further revealed their differential functions in cardiomyocyte proliferation, blocking of these pathways lead to cell-cycle arrest at different phases. Overall, our results shed light on molecular mechanism of BMP and Notch signaling pathways in cardiomyocyte proliferation during cardiac regeneration, which could lay a foundation for future development of therapeutic interventions.

## Results

### BMP signaling is activated during larval ventricle regeneration

**Figure 1:**
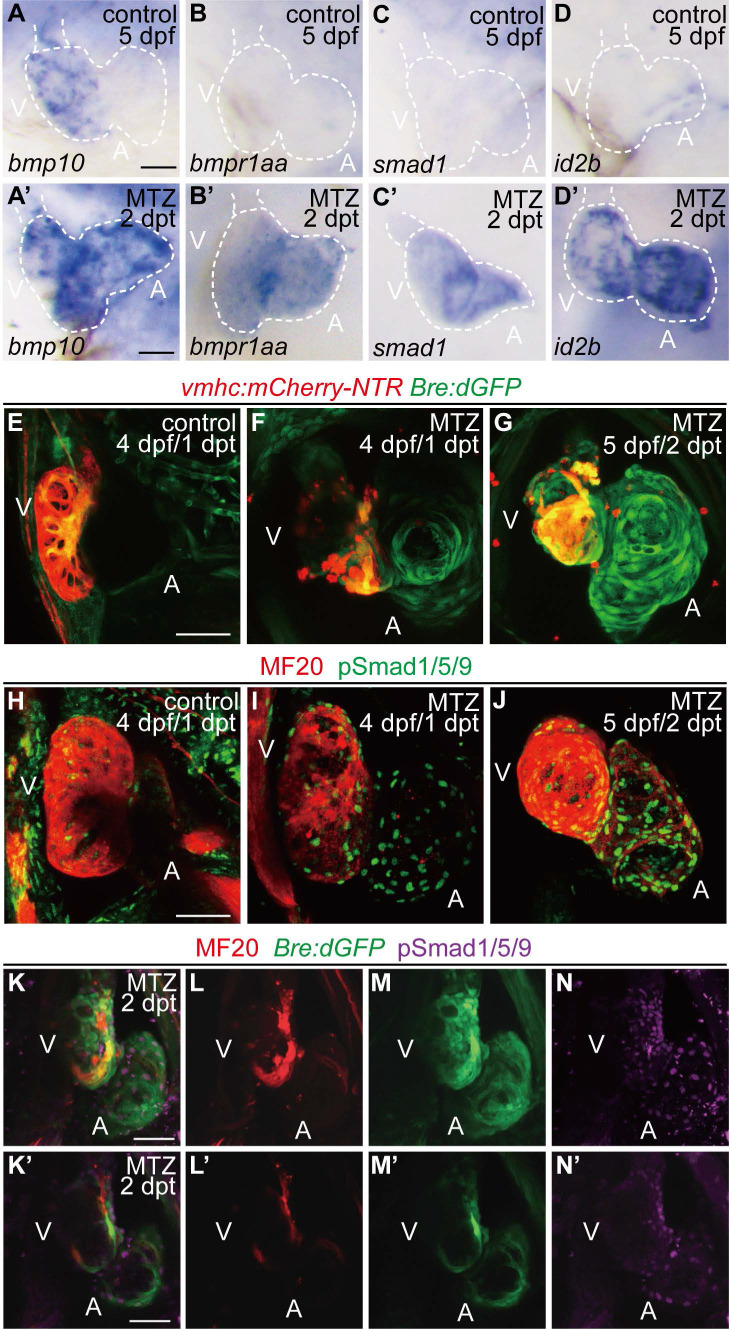
** Activation of BMP signaling pathway during ventricle regeneration. (A-D')** Whole-mount *in situ* hybridizations showed upregulated expression of BMP signaling components in ablated hearts (A'-D') compared to control hearts (A-D) at 5 dpf/2 dpt. **(E-G)** Confocal stack projections of *Tg(vmhc:mCherry-NTR; Bre:dGFP)* hearts showed BMP signaling activation at 1-2 dpt. **(H-J)** Confocal stack projections of immunofluorescence showed BMP signaling activation at 1-2 dpt. Green, phospho-Smad1/5/9; red, myosin heavy chain (MF20). **(K-N')** Overlay of Bre:dGFP and pSmad1/5/9 signals in myocardium and epicardium at 2 dpt. (K-N) stack projections, (K'-N') optical sections. Scale bars, (A-D') 25 µm, (E-N') 50 µm. dpf, days post fertilization; dpt, days post MTZ-treatment; A, atrium; V, ventricle. Dashed lines outline the hearts.

Recent research suggested that BMP signaling activation was an injury response in adult zebrafish hearts 21. To investigate the role of BMP signaling in the larval ventricle regeneration model, we treated the ventricle-specific genetic ablation line *Tg(vmhc:mCherry-NTR)* with metronidazole (MTZ) at 3 days post fertilization (dpf) 6. Whole-mount *in situ* hybridization (WISH) staining revealed that gene expressions of several BMP signaling components were dramatically increased in ablated hearts compared to that in control hearts at 5 dpf/ 2 days post MTZ-treatment (dpt) (Fig. 1A-D'). BMP ligand *bmp10* was weakly expressed in the ventricle of control hearts, probably due to the trabeculae formation at this stage. After ablation, *bmp10* expression was strongly induced, especially in the atrium (Fig. 1A, A'). Expression of BMP receptor *bmpr1aa* and signal transducer* smad1* was also up-regulated in ablated hearts, while expression of BMP signaling effector *id2b* showed a much dramatic increment after ablation (Fig. 1B-D').

To visualize the transient activation of BMP signaling in regenerating hearts, we used a reporter line *Tg(Bre:dGFP)* which expressed destabilized GFP in BMP-activated cells 30, 31. Control *Tg(vmhc:mCherry-NTR; Bre:dGFP)* larvae displayed weak GFP signal in the ventricle at 4 dpf, besides other extracardiac signals. After ventricle injury, BMP signaling was activated in the ablated heart, mainly in the atrium, at 1 dpt. The signal intensity was gradually enhanced in the atrium and extended to the ventricle at 2 dpt (Fig. 1E-G). The activation of BMP signaling during ventricle regeneration was also confirmed by immunofluorescence staining of another marker, phosphorylation of Smad1/5/9 (pSmad1/5/9), which showed a similar pattern as Bre:dGFP (Fig. 1H-J). To further validate these results, we also performed immunostaining of pSmad1/5/9 in *Tg(vmhc:mCherry-NTR; Bre:dGFP)* larvae. The results suggested that BMP signaling was strongly activated in myocardium during ventricle regeneration, while a weaker epicardial BMP signal could be detected occasionally (Fig. 1K-N'). Overall, our results confirmed that BMP signaling was activated during larval ventricle regeneration.

### BMP signaling acts downstream of Notch signaling during ventricle regeneration

**Figure 2:**
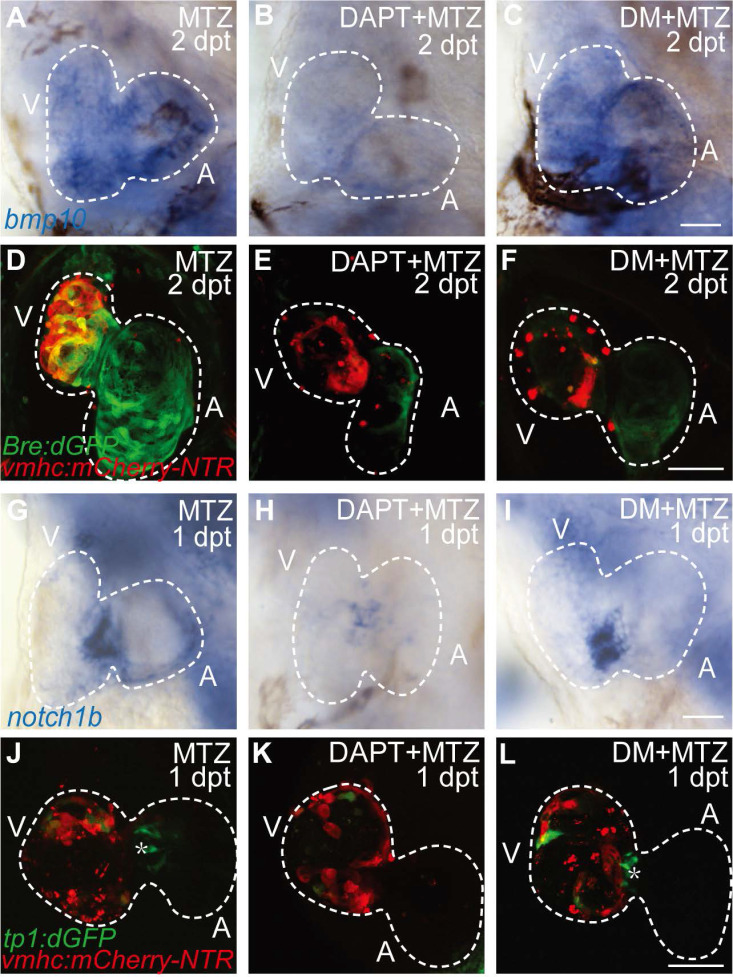
** BMP signaling acts downstream of Notch signaling during ventricle regeneration. (A-C)** Whole-mount *in situ* hybridizations showed upregulated *bmp10* expression in ablated hearts at 2 dpt could be blocked by Notch signaling inhibitor DAPT but not BMP signaling inhibitor DM. **(D-F)** Confocal stack projections of *Tg(vmhc:mCherry-NTR; Bre:dGFP)* hearts showed BMP signaling activation at 2 dpt could be blocked by both DAPT and DM. **(G-I)** Whole-mount *in situ* hybridizations showed upregulated *notch1b* expression in ablated hearts at 1 dpt could be blocked by DAPT but not DM. **(J-L)** Confocal stack projections of *Tg(vmhc:mCherry-NTR; tp1:dGFP)* hearts showed Notch signaling activation at 1 dpt could be attenuated by DAPT but not DM. Asterisk, Notch signal at the atrioventricular canal. Scale bars, (A-C, G-I) 25 µm, (D-F, J-L) 50 µm. dpt, days post MTZ-treatment; A, atrium; V, ventricle; DM, dorsomorphin. Dashed lines outline the hearts.

Previous study showed post-injury upregulation of *bmp10* expression was blocked after Notch signaling inhibition 29, which suggested that BMP signaling was regulated by Notch signaling during regeneration. Whether BMP signaling regulated Notch signaling in a reciprocal manner remained unclear. We used pharmacological approach to study the epistatic relationship between these two signaling pathways. Inhibition of Notch signaling by DAPT treatment significantly reduced *bmp10* expression in ablated hearts at 2 dpt as revealed by WISH staining (Fig. 2A, B). BMP signaling activation was also blocked as revealed by diminished Bre:dGFP signals in DAPT-treated ablated hearts (Fig. 2D, E). Treatment of BMP signaling inhibitor Dorsomorphin (DM) blocked downstream transduction of BMP pathway, but had no effect on ligand expression (Fig. 2C, F). On the other hand, WISH analysis of *notch1b* expression in ablated hearts at 1 dpt showed no difference between DM-treated group and control group, while *notch1b* upregulation was attenuated after DAPT treatment (Fig. 2G-I). We observed a similar pattern in the Notch signaling reporter line *Tg(tp1:dGFP)* that inhibition of BMP signaling by DM treatment did not affect the activation of Notch signaling in the endocardium around atrioventricular canal (Fig. 2J-L, asterisk). Thus, our results suggested that BMP signaling acted epistatic downstream of Notch signaling during ventricle regeneration.

### BMP and Notch signaling pathways differentially influence ventricle regeneration and cardiogenic factor re-activation

**Figure 3:**
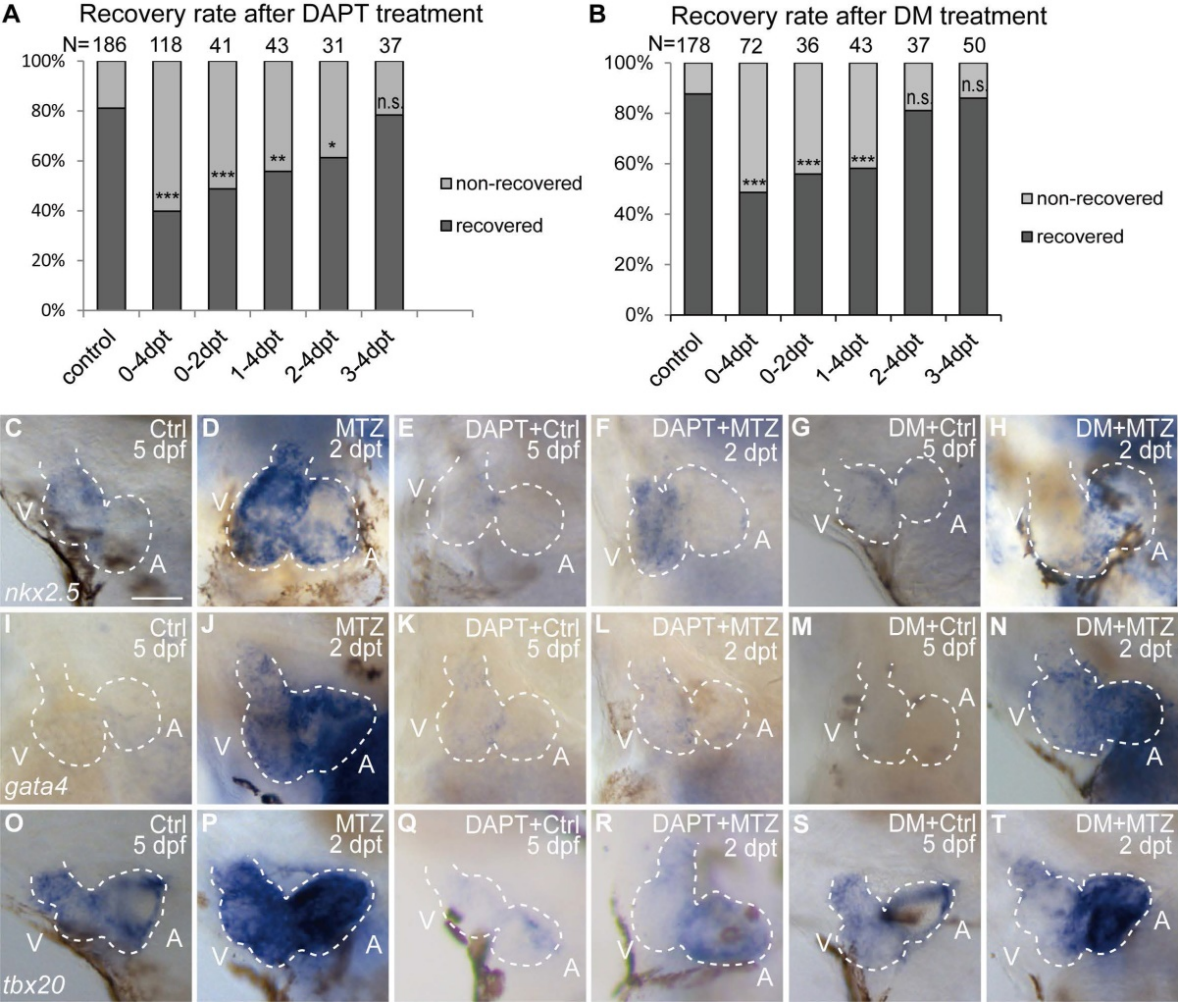
** BMP and Notch signaling pathways differentially influence ventricle regeneration and cardiogenic factor re-activation. (A, B)** Quantification of heart recovery rate in control and DAPT-treated or DM-treated ablated hearts at 4 dpt. The numbers of larvae analyzed were indicated. Binomial test, n.s, non-significant, *, *P* < 0.05, **, *P* <0.01, ***, *P* < 0.001. **(C-T)** Whole-mount *in situ* hybridizations showed different effects on the re-activation of cardiogenic factor *nkx2.5* (C-H), *gata4* (I-N), *tbx20* (O-T) after DAPT or DM treatment at 5 dpf/2 dpt. Scale bar, 50 µm. dpf, days post fertilization; dpt, days post MTZ-treatment; A, atrium; V, ventricle; Ctrl, control; DM, dorsomorphin. Dashed lines outline the hearts.

To compare the functions of Notch and BMP signaling in ventricle regeneration, we first quantified the heart recovery rate at 4 dpt. DAPT treatment during the whole course (0-4 dpt) or early stage (0-2 dpt) of ventricle regeneration significantly reduced the heart recovery rates (Fig. 3A, 39.8%, N=118, and 48.8%, N=41, respectively) compared to control group (81.2%, N=186), which was in accordance with previous report 6, 32. A similar pattern of reduced heart recovery rate was observed when DM treatment was applied during 0-4 dpt (Fig. 3B, 48.6%, N=72 vs 87.6%, N=178 in control group) and 0-2 dpt (55.9%, N=36). We further assessed the effects of pathway inhibition during late stage of ventricle regeneration. DAPT treatment during 1-4 dpt and 2-4 dpt also decreased the heart recovery rates, though to a lesser degree of reduction (Fig. 3A, 55.8%, N=43, and 61.3%, N=31, respectively), while DAPT treatment during 3-4 dpt did not obviously change the recovery rate (78.4%, N=37). On the contrast, DM treatment showed reduction effect only when applied during 1-4 dpt (Fig. 3B, 58.1%, N=43) but not during 2-4 dpt (81.1%, N=37) or 3-4 dpt (86.0%, N=50). Such difference between DAPT and DM treatments indicated that BMP and Notch signaling pathways influence ventricle regeneration in different time windows.

We then examined the effect of Notch or BMP signaling blockage on re-activation of early cardiogenic transcription factors. Expression of *nkx2.5*, *gata4* and *tbx20* was dramatically increased in ablated hearts compared to control hearts at 2 dpt as revealed by WISH experiments (Fig. 3C, D, I, J, O, P). After DAPT treatment, cardiogenic factor re-activation was blocked in both chambers (Fig. 3E, F, K, L, Q, R) similar as previously reported 6, 29. However, DM treatment showed heterogeneous effect on cardiogenic factor re-activation in the two chambers, with an apparent stronger reduction in the ventricle (Fig. 3G, H, M, N, S, T). These results suggested BMP and Notch signaling pathways might have differential functions in cardiogenic factor re-activation.

### FUCCI as a cardiomyocyte proliferation indicator during ventricle regeneration

**Figure 4:**
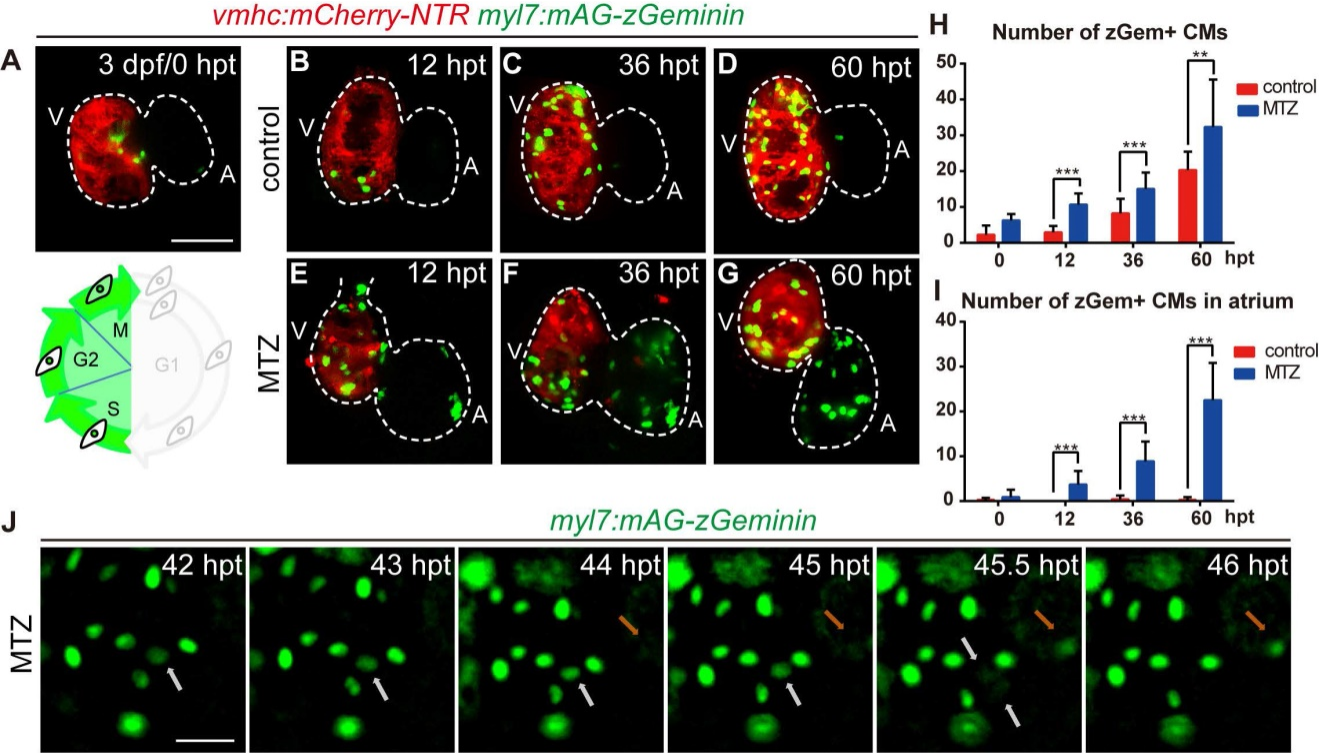
** FUCCI as a cardiomyocyte proliferation indicator during ventricle regeneration. (A-G)** Confocal stack projections of *Tg(vmhc:mCherry-NTR; myl7:mAG-zGeminin)* hearts showed pattern of zGem+ CMs at 3 dpf/0 hpt, which are in the S, G2 and M phases of the cell-cycle (A). Numbers of zGem+ CMs dramatically increased in the atrium of ablated hearts (E-G) compared to control hearts (B-D). **(H, I)** Quantification of zGem+ CM numbers in the whole heart (H) or in the atrium only (I) of control and ablated groups at 0, 12, 36 and 60 hpt. Mean + SD, N=8-15 for each time point, Student's t-test, **, *P* <0.01, ***, *P* < 0.001. **(J)** Time-lapse images of *Tg(myl7:mAG-zGeminin)* hearts showed the dynamic pattern of zGem+ CMs during 42-46 hpt. White arrows point to a CM which lost zGem+ signal after division, while orange arrows point to a CM with newly appeared zGem+ signal. Scale bars, (A-G) 50 µm, (J) 20 µm. dpf, days post fertilization; hpt, hours post MTZ treatment; A, atrium; V, ventricle; CM, cardiomyocyte. Dashed lines outline the hearts.

Next, we focused on the regulation of cardiomyocyte proliferation, which is an important aspect of heart regeneration 33, 34. FUCCI was developed as a tool to monitor cell-cycle behavior *in vivo* 35-37. To visualize the dynamic process of cardiomyocyte proliferation, we crossed cardiomyocyte-specific FUCCI line *Tg(myl7:mAG-zGeminin)* with *Tg(vmhc:mCherry-NTR)* so that cardiomyocytes in S/G_2_/M phases would be labelled with green fluorescence (Fig. 4A). Few zGem+ cardiomyocytes were observed in control hearts at 3 dpf, mainly in the ventricle. The number of zGem+ cardiomyocytes gradually increased (Fig. 4A-D), probably due to trabeculae formation in the ventricle at this stage. After MTZ treatment zGem+ cardiomyocytes first showed up in the atrium of ablated hearts at 12 hours post treatment (hpt) with increasing numbers during regeneration (Fig. 4E-G). Quantification of zGem+ cardiomyocyte numbers at 0, 12, 36, 60 hpt indicated that myocardial proliferation in ablated hearts was enhanced (6.2 ± 1.8, 10.7 ± 3.1, 15.1 ± 4.5, 32.3 ± 13.3, N=8, 15, 15, 10, respectively) compared to control hearts (2.2 ± 2.6, 2.9 ± 1.8, 8.2 ± 4.1, 20.3 ± 5.1, N=8, 15, 13, 12, respectively) (Fig. 4H). Consistent with this pattern, the atrium responded to ventricle injury and the quantity of zGem+ cardiomyocytes in the atrium of ablated hearts escalated markedly (0.9 ± 1.6, 3.7 ± 3.0, 8.9 ± 4.4, 22.5 ± 8.3, N=8, 15, 15, 10, respectively) compared to control atriums (0.2 ± 0.5, 0.0 ± 0.0, 0.4 ± 0.9, 0.2 ± 0.6, N=8, 15, 13, 12, respectively) (Fig. 4I). Short period time-lapse fluorescence imaging captured how FUCCI system reflected the cell cycle state with loss of zGem+ signals after cell division (Fig. 4J, white arrows), as well as newly appeared zGem+ signal suggesting the cardiomyocyte just entered the proliferating stage (orange arrows).

### Inhibition of BMP and Notch signaling pathways differentially suppresses FUCCI expression

**Figure 5:**
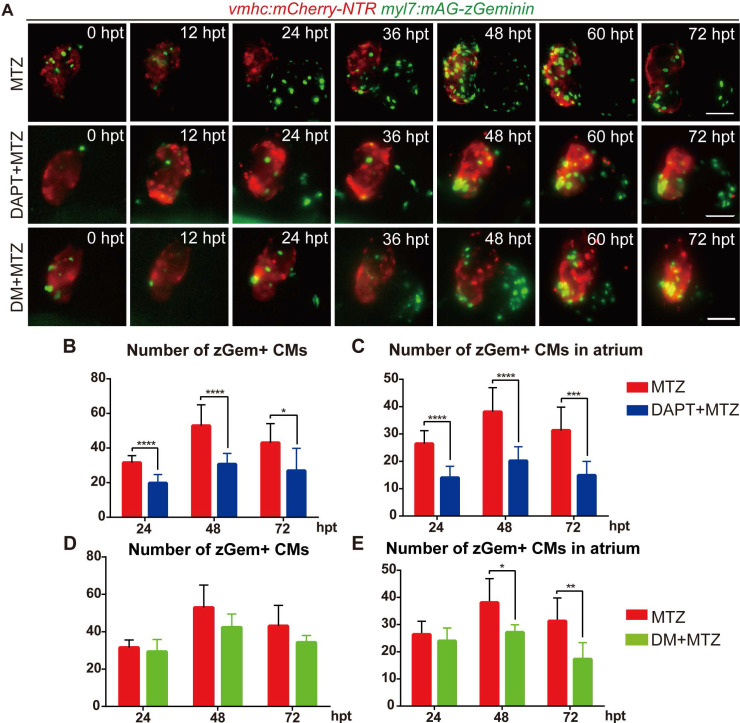
** Inhibition of BMP and Notch signaling pathways differentially suppresses FUCCI expression. (A)** Time-lapse images of *Tg(vmhc:mCherry-NTR; myl7:mAG-zGeminin)* hearts showed differential patterns of zGem+ CMs in ablated, DAPT-treated ablated and DM-treated ablated groups at 0-72 hpt. Scale bars, 50 µm. hpt, hours post MTZ-treatment; CM, cardiomyocyte; DM, dorsomorphin. **(B, C)** Quantification of zGem+ CM numbers in the whole heart (B) or in the atrium only (C) of ablated and DAPT-treated ablated groups at 24, 48 and 72 hpt. Mean + SD, N=8-15 for each group, Student's t-test, *, *P* <0.05, ***, *P* < 0.001, ****, *P* < 0.0001. **(D, E)** Quantification of zGem+ CM numbers in the whole heart (D) or in the atrium only (E) of ablated and DM-treated ablated groups at 24, 48 and 72 hpt. Mean + SD, N=6-13 for each group, Student's t-test, *, *P* <0.05, **, *P* < 0.01.

To further explore the functions of BMP and Notch signaling in cardiomyocyte proliferation, we performed pharmacological inhibition experiments in *Tg(vmhc:mCherry-NTR; myl7:mAG-zGeminin)* and recorded the change in zGem+ cardiomyocyte pattern by 12-hour-interval living imaging (Fig. 5A). Compared with control hearts, the zGem+ signal decreased in DAPT-treated ablated hearts from 24 hpt, which was more obvious in the atrium. DM treatment showed a lesser inhibitory effect on zGem+ signals. We then quantified the numbers of zGem+ cardiomyocytes in the ablated hearts at 24, 48, 72 hpt. DAPT treatment significantly reduced the numbers from 31.6 ± 4.1 (N=9) to 19.8 ± 4.9 (N=13) at 24 hpt, from 52.9 ± 12.0 (N=11) to 30.7 ± 6.2 (N=10) at 48 hpt, and from 43.0 ± 11.1 (N=9) to 26.9 ± 13.0 (N=8) at 72 hpt (Fig. 5B). Considering the numbers of zGem+ cardiomyocytes in the atrium only, we found a similar trend upon DAPT treatment that the numbers dropped from 26.4 ± 4.8 (N=9) to 14.0 ± 4.2 (N=13) at 24 hpt, from 38.1 ± 8.9 (N=11) to 20.2 ± 5.2 (N=10) at 48 hpt, and from 31.3 ± 8.5 (N=9) to 14.9 ± 5.1 (N=8) at 72 hpt (Fig. 5C). However, the numbers of zGem+ cardiomyocytes did not show statistically significant reduction upon DM treatment in the whole hearts at different timepoints with the only differences observed in the atrial zGem+ signals at 48 hpt (38.1 ± 8.9 vs. 27.2 ± 2.8, N=11, 6, respectively) and 72 hpt (31.3 ± 8.5 vs. 17.3 ± 6.0, N=9, 6, respectively) (Fig. 5D, E). In short, these results suggested BMP and Notch signaling might have differential functions in cardiomyocyte proliferation.

### BMP and Notch signaling pathways differentially influence cardiomyocyte cell-cycle progression

**Figure 6:**
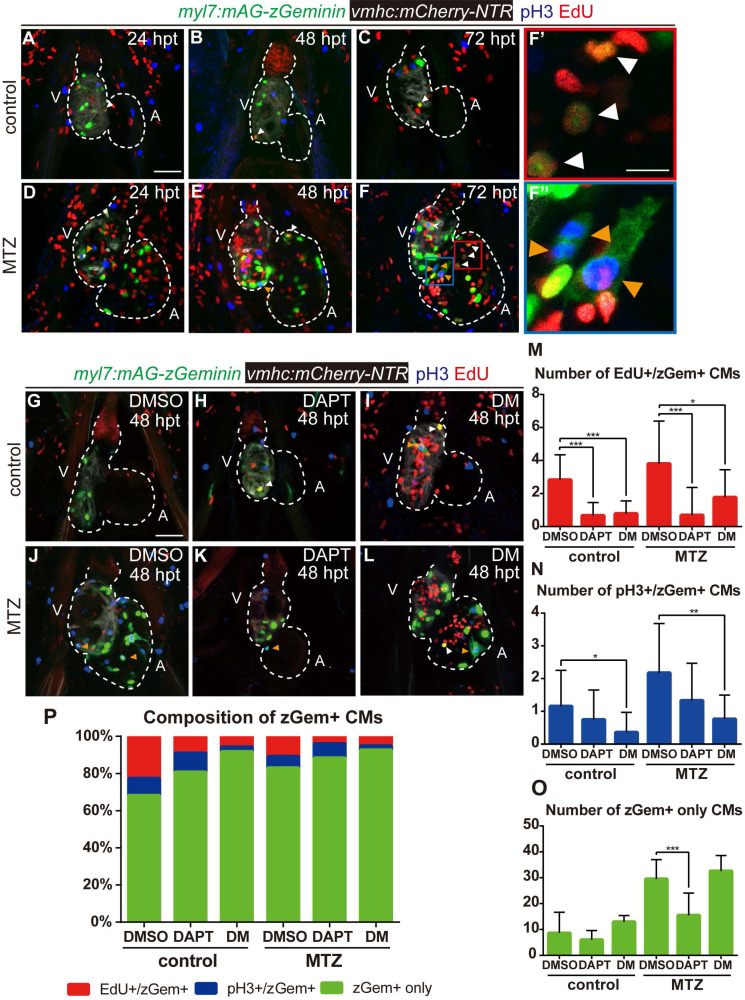
** BMP and Notch signaling pathways differentially influence cardiomyocyte cell-cycle progression. (A-F'')** Confocal stack projections of *Tg(vmhc:mCherry-NTR; myl7:mAG-zGeminin)* hearts with immunofluorescence of phospho-histone H3 and EdU showed proliferating CMs in different phases of cell cycle in control or ablated groups at 24, 48 and 72 hpt. (F', F”) magnified red and blue box areas in F. White arrowheads point to EdU+/ zGem+ CMs, orange arrowheads point to pH3+/ zGem+ CMs. **(G-L)** Confocal stack projections of *Tg(vmhc:mCherry-NTR; myl7:mAG-zGeminin*) hearts with immunofluorescence of phospho-histone H3 and EdU in control or ablated groups without or with DAPT or DM treatment at 48 hpt. White arrowheads point to EdU+/ zGem+ CMs, orange arrowheads point to pH3+/ zGem+ CMs. **(M-P)** Quantification of EdU+/zGem+ (M), pH3+/zGem+ (N), and zGem+ only (O) CM number in control or ablated groups without or with DAPT or DM treatment at 48 hpt. The respective proportions in total zGem+ CMs were shown in P. Mean + SD, N=12-18 for each group, Student's t-test, *, *P* < 0.05, **,* P* < 0.01, ***, *P* < 0.001. Scale bars, (A-L) 50 µm, (F', F”) 20 µm. hpt, hours post MTZ-treatment; A, atrium; V, ventricle; CM, cardiomyocyte; DM, dorsomorphin. Dashed lines outline the hearts.

Previous studies reported that both BMP and Notch signaling regulated cardiomyocyte proliferation 29. To resolve the discrepancy in the zGem+ signals observed above, we examined other proliferation markers, such as immunostaining of phospho-histone H3 (referred as pH3) and EdU pulsed labelling, in *Tg(vmhc:mCherry-NTR; myl7:mAG-zGeminin)* fish. The overlapped signals of pH3 and EdU with zGem+ could help to eliminate the interference caused by proliferative cells in adjacent tissues, such as endocardium, epicardium and blood cells. Proliferating cardiomyocytes were in different cell-cycle stages (Fig. 6A-F''), an EdU+/zGem+ cell represented a cardiomyocyte at S phase (white arrowheads), a pH3+/zGem+ cell represented a cardiomyocyte at M phase (orange arrowheads), while a zGem+ only cell without pH3 or EdU signals was considered in G2 phase. We then assessed the marker distribution in fish treated with DAPT or DM at 48 hpt (Fig. 6G-L) and quantified the numbers of proliferating cardiomyocytes in different cell-cycle phases (Fig. 6M-O). In terms of BMP signaling inhibition, DM treatment reduced the numbers of EdU+/zGem+ cells in control hearts (2.8 ± 1.5 vs. 0.8 ± 0.8, N=12, 14, respectively) and ablated hearts (3.8 ± 2.6 vs. 1.8 ± 1.7, N=16, 13, respectively). Similarly, the numbers of pH3+/zGem+ cells decreased in control hearts (1.2 ± 1.1 vs. 0.4 ± 0.6, N=13, 14, respectively) and ablated hearts (2.2 ± 1.5 vs. 0.8 ± 0.8, N=17, 17, respectively) upon DM treatment. However, the numbers of zGem+ only cells remained unchanged between groups with or without DM treatment. On the contrast, Notch signaling inhibition with DAPT treatment significantly reduced the number of zGem+ only cells in the ablated heart (29.5 ± 7.5 vs. 15.5 ± 8.6, N=18, 17, respectively), reflecting a significant reduction in the number of G2 phase cardiomyocytes. DAPT treatment also reduced the numbers of EdU+/zGem+ cells in control hearts (2.8 ± 1.5 vs. 0.7 ± 0.8, N=12, 15, respectively) and ablated hearts (3.8 ± 2.6 vs. 0.7 ± 1.7, N=16, 16, respectively). The reduction in the numbers of pH3+/zGem+ cells upon DAPT treatment was not statistically significant. We also plotted the data based on the proportions of marked cardiomyocytes in different cell-cycle phases (Fig. 6P). Reduced percentages of cardiomyocytes in S and M phases and increased percentage of G2 phase cardiomyocytes implied that DM treatment may cause cell-cycle arrest at G2 phase. Blocking Notch signaling pathway may possibly led to G0/G1 phase arrest because of the universally dwindled number of proliferative cardiomyocytes. Taken together, our results suggested that BMP and Notch signaling pathways regulated myocardium proliferation through differentially influence cardiomyocyte cell-cycle progression.

## Discussion

In this study we affirmed the upregulated expression of multiple BMP signaling components by WISH and the activation of BMP signaling in the myocardium and epicardium by pSmad1/5/9 immunostaining and Bre:dGFP reporter signals during larval ventricle regeneration. In zebrafish larvae, *bmp10* has been reported to express in the endocardium, while *bmp10l* is expressed in the myocardium 38, 39. The expression of *bmp6* and *id2b* is endocardial specific during heart regeneration 40. Wu et al. reported that *bmp2b* and *bmp7* was expressed in the endocardium and epicardium of cryo-injured hearts, while pSmad1/5/9 signals were up-regulated in the myocardium 21. Thus, we speculate that BMP signaling activation in myocardium may be promoted by enhanced ligand secretion from the endocardium after ventricle ablation, probably through interaction with other endocardial signaling pathways 26, 29, 41.

It is suggested that multiple signaling pathways eventually converge together and control cardiomyocyte cell cycle progression through certain function axes 42, implying the importance to understand the complex crosstalk among different signaling pathways. Cardiomyocyte proliferation in injured hearts with defective endocardial Notch signaling can be partially restored by WNT antagonist treatment, demonstrating the crosstalk between Notch and WNT/β-catenin signaling 43. Moreover, endocardial Notch signaling promotes cardiomyocyte reprogramming and cardiac regeneration through activating myocardial Erbb2 and BMP signaling 29. Our results proved that inhibition of Notch signaling by DAPT abolished the upregulated expression of ligand *bmp10* and blocked BMP signaling activation. BMP pathway blockage by DM treatment did not reciprocally affect expression of *notch1b* nor signals of Notch signaling reporter, suggesting BMP signaling acts epistatic downstream of Notch signaling. Whether this regulation is cell autonomous or not requires further investigation. Our results showed a broader expression of *bmp10* by WISH than the more confined Notch signaling revealed by tp1:dGFP fluorescence, suggesting at least certain cell non-autonomous mechanism is involved in this process. Interestingly, we also observed differential influence of Notch and BMP signaling on the re-activation of cardiogenic factors during regeneration. Bmp signaling exerts opposite effects on chamber differentiation during early heart development 44, 45, so one possible explanation is that BMP pathway regulates this process in a chamber-specific way by which the ventricle, instead of the atrium, is more susceptible. Another possibility is that the BMP pathway may not be directly involved in the regulation of cardiogenic factor re-activation, but rather that its inhibition on cardiomyocyte proliferation leads to a decrease in the number of ventricular cardiomyocytes as well as the expression level of cardiogenic factors. This is consistent with a previous report that BMP10 is responsible for cardiomyocyte proliferation while NRG1 regulates cardiomyocyte differentiation downstream of Notch signaling during mice ventricle trabeculation 27.

Many studies on the regulation of cell proliferation focus on the change in cell cycle progression 42, 46, 47. Blocking Notch signaling pathway has been reported in several models to cause G0/G1 phase arrest by influencing *p27^Kip1^* and other elements, thereby affecting proliferation 48-50. On the contrary, few studies have been conducted on the mechanism of BMP pathway in regulating proliferative events. Overexpression of Id1 increases Cdk4 levels and reduces *p21^Clip1^*, thus promoting cell cycle progression in mouse cardiomyocytes 51. BMP signaling responses to laminar shear stress by activation of p21 which leads to G2/M phase arrest of human bladder transitional carcinoma cells 52. In our larval ventricle regeneration model, inhibition of BMP signaling reduced the numbers of EdU+/zGem+ and pH3+/zGem+ cardiomyocytes but had no effect on the number of zGem+ only cardiomyocytes, implying cell-cycle arrest at G2 phase. Blocking Notch signaling pathway may possibly led to G0/G1 phase arrest because of the universally dwindled number of proliferative cardiomyocytes. Our study provides novel insights into and potential direction for the understanding of cardiomyocyte proliferation in zebrafish ventricle regeneration process. The molecular mechanisms how these signaling pathways regulate proliferation, and whether it is conserved in different species and under different situations warrants further investigation.

## Materials and methods

### Zebrafish husbandry

Zebrafish were maintained under standard conditions 53. Zebrafish embryos were raised at 28 °C and were staged according to Kimmel et al. 54. All experiments were performed in accordance with institutional and national animal welfare guidelines. The transgenic lines used in this study were as follows: *Tg(vmhc:mCherry-NTR)*,* Tg(Bre:dGFP)*,* Tg(tp1:dGFP)*,* Tg(myl7:mAG-zGeminin)*.

### *In situ* hybridization

Whole-mount *in situ* hybridization was performed as previously described 6, including *notch1b*, *nkx2*.5, *gata4*, *tbx20*. The probes for *bmp10*, *bmpr1aa*, *id2b* and *smad1* were amplified by PCR with the following primers: *bmp10* F-GCAGCCAGCAAGTAAGAGGA and R-GGTAGAGCAGGGAGATGGGA, *bmpr1aa* F-TAGCCAACCCCAATGCTTAC and R-GCCCATTTGTCTCGCAGGTAT, *id2b* F-TCGTGCCGAGTTTACCG and R-GCAATACCATACAGCTCCAGAT, *smad1* F-CACGCTTCCGTAACCCACTCC and R-GCTCAAACATTCGGCATACACCT.

### Chemical treatment

*Tg(vmhc:mCherry-NTR)* larvae were treated with 6 mM MTZ (metronidazole, Sigma) in E3 water at 3 dpf for four hours as previously described 6. After washing with fresh E3 water for three times, the larvae were then incubated in 100 μM DAPT (Sigma-Aldrich), 7.5 μM Dorsomorphin (Sigma-Aldrich) or 1% DMSO (dimethyl sulfoxide, Thermo Fisher Scientific) as control for 0-2 dpt or other time frames as indicated.

### EdU labeling

The larvae were incubated with 500 μM EdU for 1 h in E3 water with 2% DMSO to facilitate EdU solubilization. After pulse labeling, larvae were rinsed with E3 water, anaesthetized with 0.2% tricaine and fixed in 4% PFA. The CLICK-IT reaction for EdU labeling was performed according to the manufacturer's instruction (Thermo Fisher Scientific).

### Immunostaining

Immunofluorescence staining on whole-mount larvae was performed as previously described 6, using the following primary antibodies: anti-phospho-histone H3(Ser10) (rabbit; Merck Millipore, 06570), anti-phospho-Smad1/5/9 (rabbit; CST, 13820), anti-MHC (mouse; DSHB, MF20). The secondary antibodies used were Alexa Fluor 405 goat anti-rabbit IgG, Alexa Fluor 488 goat anti-rabbit IgG and Alexa Fluor 555 goat anti-mouse IgG from Thermo Fisher Scientific.

### Imaging

Live imaging and immunostaining images were obtained using a Zeiss LSM880 confocal microscope or an Olympus IX83 inverted microscope. The numbers of differentially marked cardiomyocytes were counted with ZEN software or cellSens software.

### Statistical analysis

Values were presented as mean ± SD. Statistical significance was defined as a threshold of *P* < 0.05 determined by Student's t-test between two groups, ANOVA analysis between more than two groups or Binomial test in quantification of the percentage of recovered hearts.

## Abbreviations

A: atrium
CM: cardiomyocyte
Ctrl: control
DM: Dorsomorphin
dpf: days post fertilization
dpt: days post MTZ-treatment
FUCCI: fluorescent ubiquitylation-based cell cycle indicator
hpt: hours post MTZ-treatment
MI: myocardial infarction
MTZ: metronidazole
pH3: phospho-histone H3
pSmad1/5/9: phospho-Smad1/Smad5/Smad9
V: ventricle
WISH: whole-mount *in situ* hybridization
